# Tax awareness and perceived cost of sugar-sweetened beverages in four countries between 2017 and 2019: findings from the international food policy study

**DOI:** 10.1186/s12966-022-01277-1

**Published:** 2022-03-31

**Authors:** Rachel B. Acton, Lana Vanderlee, Jean Adams, Sharon I. Kirkpatrick, Lilia S. Pedraza, Gary Sacks, Christine M. White, Martin White, David Hammond

**Affiliations:** 1 School of Public Health Sciences, University of Waterloo, 200 University Ave W, Waterloo, ON N2L 3G1 Canada; 2 École de Nutrition, Centre de Nutrition, Santé Et Société (NUTRISS), Université Laval, 2425 rue de L’Agriculture, Québec, QC G1V 0A6 Canada; 3 Centre for Diet and Activity Research (CEDAR), MRC Epidemiology Unit, School of Clinical Medicine, Institute of Metabolic Science, University of Cambridge, Cambridge Biomedical Campus, Box 285, Cambridge, CB2 0QQ UK; 4 Center for Nutrition and Health Research, Instituto Nacional de Salud Pública, Col. Santa María Ahuacatitlán, Cerrada los Pinos Y Caminera, Av. Universidad 655, C.P. 62100 Morelos, Mexico; 5 Global Obesity Centre, Deakin University, Melbourne Burwood Campus, Burwood, VIC 3125 Australia

**Keywords:** Sugar-sweetened beverages, Tax, Nutrition policy, Awareness, Perceived cost

## Abstract

**Background:**

The public health benefits of sugar-sweetened beverage (SSB) taxes often rely on, among other things, changes to consumer purchases. Thus, perceived cost of SSBs and signalling effects—via awareness of the tax—may impact the effectiveness of SSB taxes on consumer purchases.

**Objective:**

The study sought to examine perceived cost of SSBs, tax awareness, and changes in beverage purchasing over time and across four countries with and without SSB taxes.

**Methods:**

The study used data from the 2017, 2018 and 2019 waves of the International Food Policy Study. Annual cross-sectional online surveys were conducted in Australia, Mexico, UK and US, which captured perceived cost of SSBs relative to non-SSBs in all countries (with Australia as a no-tax comparator), and measures of tax awareness and participants’ reported changes in beverage purchasing in response to SSB taxes in Mexico (tax implemented in 2014), UK (tax implemented in 2018) and US (subnational taxes since 2015). Logistic regression models evaluated the measures across years and socio-demographic groups.

**Results:**

Perceived cost of SSBs relative to non-SSBs was higher in Mexico (all three years) and the UK (2018 and 2019 following tax implementation) than Australia and the US. Tax awareness was higher in UK than Mexico, and decreased over time among Mexican respondents. Patterns of reported beverage purchasing changes in response to the tax were similar across Mexico, UK and US, with the largest changes reported by Mexican respondents. Respondents with characteristics corresponding to lower socioeconomic status were less likely to be aware of an SSB tax, but more likely to perceive SSBs to cost more than non-SSBs and report changes in purchasing in response to the tax, where there was one.

**Conclusions:**

This study suggests that in countries where a national SSB tax was present (Mexico, UK), perceived cost of SSBs and tax awareness were higher compared to countries with no SSB tax (Australia) or subnational SSB taxes (US), respectively, and suggests that perceived cost and tax awareness represent distinct constructs. Improving the ‘signalling effect’ of existing SSB taxes may be warranted, particularly in tax settings where consumer behaviour change is a policy objective.

**Supplementary Information:**

The online version contains supplementary material available at 10.1186/s12966-022-01277-1.

## Introduction

Sugar-sweetened beverages (SSBs) remain a prominent target for public health interventions due to their contribution to sugar and energy intake and association with increased risk of type 2 diabetes, obesity, heart disease, dental caries, and other obesity-related diseases [1]. Consequently, there is a growing interest in many countries for strategies to reduce SSB consumption at the population level [2].

One strategy for reducing SSB consumption is taxation. Over 40 countries and cities have implemented SSB taxes [3], which aim to reduce purchasing and consumption of the targeted beverages, incentivize product reformulation by the beverage industry, and/or generate revenue, which is often reinvested towards other public health endeavors. Observational evidence to date from evaluations of national and city-level SSB taxes suggests that these policies can be effective at one or more of the following: increasing prices [4–11]; reducing purchasing and consumption [6–9, 12–23]; and encouraging the beverage industry to reformulate the sugar content of their beverage offerings [24–27].

There is, however, substantial variability in the magnitude of impacts observed in relation to existing SSB taxes, which may be largely explained by differences in tax design. The majority of SSB taxes implemented to date are excise taxes that are levied on the manufacturer and may or may not be intended to be passed down to the consumer. Most taxes apply volume-specific price increases to qualifying SSB products (e.g., 1 peso/litre in Mexico, or $0.01–0.02/fl oz in city-level taxes in the United States (US)) [28]. In contrast, other excise SSB taxes—such as the soft drinks industry levy (SDIL) in the United Kingdom (UK)—utilize a sugar-specific design, which assigns ‘tiered’ or continuous price increases based on the sugar content of beverages, and tend to emphasize product reformulation on the part of the manufacturer rather than consumer behaviour change [29, 30]. Given the differences in design and policy objectives across SSB taxes, some taxes may rely to a greater extent on certain mechanisms of action than others, such as price responsiveness, signalling via awareness of the tax, and reformulation.

The most common mechanism of SSB and other public health taxes is the economic theory that as the price of a good increases, demand for that good decreases [31]. Evidence suggests that consumers respond to price increases whether or not they are aware of the tax itself [32], and that their response may be conscious or unconscious [33]. A recent meta-analysis summarized that consumers reduce their SSB purchases by an average of 10% when SSB prices are raised by 10% [18]. However, price responsiveness varies across different consumer groups. For example, individuals who consume SSBs more frequently tend to be more responsive to SSB taxes, as do consumers of lower income [21, 31, 34]. The extent to which an excise tax is passed on to consumers is also an important factor determining how consumers respond to the price increases: estimates of pass-through rates from real-world SSB taxes range from less than 50% to over 100% pass-through [35].

Evidence also suggests that the ‘signalling effects’ of an SSB tax may play an important role in changing consumer behaviour, independent of price effects [36–38]. Signalling effects refer to the ways in which the implementation of a tax may signal to consumers that consumption of the taxed goods should be reduced, beyond the mechanism of price increases [38]. Signals may be implicit (e.g., the fact that the government is taxing a product may suggest to consumers that consumption should be reduced; related to the ‘expressive function of law’ [39]), or explicit (e.g., when governments communicate specific information on the health harms associated with the taxed products) [40]. Scant research has explored signalling effects in the context of SSB taxes, and has predominantly focused on the explicit impacts of health communication campaigns, political debate, and media attention. Two studies, one evaluating soda sales in Berkeley, US [41] and another surveying a cross-sectional sample of South African adults [42], found that soda sales dropped and knowledge of the health harms of SSBs and intention to reduce consumption increased, respectively, *prior* to tax implementation, suggesting that media coverage and pro-tax campaigns in these jurisdictions had impacts beyond that of price.

Evidence on consumer awareness of SSB taxes can also provide insight into potential signalling effects of SSB taxes, given that awareness is required for signalling effects to occur. Overall, observational studies assessing awareness of existing SSB taxes demonstrate heterogeneity within and across jurisdictions. A study assessing adults in Mexico found that 65% reported being aware of the national SSB tax of 1 peso/L approximately two years post-implementation [36], whereas in focus groups involving adolescents in north-west Mexico, the majority of respondents were not aware of the SSB tax [43]. In South Africa, focus groups among adults three months prior to implementation of their national SSB tax (ZAR 2.1 cents/g sugar) found the majority of participants were not aware of the upcoming tax. [44]

Lastly, some SSB taxes—such as the UK’s SDIL—are designed by a governing body with the primary intention of encouraging product reformulation by targeting beverage manufacturers directly, with no explicit intention of influencing consumer behaviour [45]. In such cases, neither price effects nor signalling effects are primary policy objectives, but may nonetheless impact consumer behaviour. For example, recent evaluations in the UK have suggested that the introduction of the tiered levy has prompted substantial reformulation of sugary drinks by the beverage industry, with an increasing number of lower-calorie options being introduced [24, 46]. And although consumer perceptions and behaviour were not explicitly targeted by the SDIL, a recent cross-sectional analysis of parents in the UK found that 92% of respondents reported being aware of the SDIL, and 41% reported an intention to reduce their family’s SSB consumption following the levy introduction [47].

Given the differences in mechanisms relied upon by existing SSB taxes and the apparent heterogeneity of price responsiveness and tax awareness across settings, comprehensive assessments of such measures across jurisdictions are warranted. Evaluating consumers’ perceived cost of SSBs and awareness of SSB taxes may help to increase our understanding of how price and signalling effects contribute to consumer purchasing behaviour in settings with different SSB tax designs. While there is an established link between price perceptions and purchase intention and behaviour [48], to our knowledge, no published studies have compared measures of price perceptions or tax awareness over time or across multiple countries with and without SSB taxes. In addition, research has seldom examined consumers’ self-reported changes in purchasing behaviour in response to such taxes, which could help to complement existing evidence from sales data, particularly in settings where consumer behaviour change is not a primary policy objective and other mechanisms (i.e., reformulation) may be more impactful.

In this study we use data from the International Food Policy Study (IFPS), which performs annual repeat cross-sectional surveys in Australia, Mexico, the UK and the US. The IFPS provides a unique opportunity to examine measures of perceived SSB cost, tax awareness and self-reported impacts over time and across multiple countries. National SSB taxes were implemented in Mexico and the UK in January 2014 and April 2018, respectively, and seven city-level taxes in the US (Albany CA, Berkeley CA, Boulder CO, Oakland CA, Philadelphia PA, San Francisco CA, Seattle WA) have been implemented and upheld since 2015. Australia, where no SSB tax has been implemented, was included as a comparator.

In this study, we aimed to examine three primary measures: 1) perceived cost of SSBs (relative to non-SSBs) over time in Australia, Mexico, the UK and the US; 2) self-reported awareness of SSB taxes in years where taxes were present in Mexico, the UK and the US; and 3) self-reported changes in beverage purchases in response to SSB taxes in years where taxes were present, and whether or not this was influenced by perceived cost of drinks with sugar. Potential associations between the outcomes of interest and socio-demographic variables were also explored.

## Methods

We analysed data from the IFPS 2017, 2018 and 2019 waves. Online surveys were conducted in December 2017 (*N* = 16,739), November–December 2018 (*N* = 18,427), and November–December 2019 (*N* = 16,861) in Australia, Mexico, the UK, and the US. Full methods of the IFPS surveys are reported elsewhere [49]. The study was reviewed by and received ethics clearance through a University of Waterloo Research Ethics Committee (ORE #30,829).

### Sample recruitment

The IFPS samples were recruited through Nielsen Consumer Insights Global Panel, using a standardized recruitment sampling strategy employing both probability and non-probability sampling methods across countries. Quotas for age and sex were applied to facilitate recruitment of a diverse sample that approximated the known proportions in each country for males and females across age groups [49–51].

Eligibility criteria included being 18–64 (2017) or ≥ 18 years of age (2018–2019) and residing in a target country. Email invitations with a unique link were sent to a random sample of panelists that met inclusion criteria. If deemed eligible, potential respondents were provided with information about the study and provided consent prior to participating. Surveys were conducted in the primary language(s) spoken in each country. Respondents received remuneration in accordance with their panel’s usual incentive structure. A data integrity check was included part way through the survey, and additional data integrity analyses were conducted during data cleaning.

Sampling weights.

Post-stratification sample weights were constructed each year for each country separately based on known population totals by age, sex at birth, region, ethnicity (except in 2017), and education (except in Mexico and 2017) [49–51].

### Survey measures

The main outcome measures used in this study were adapted from traditional tax and price measures used in fields outside of nutrition, such as tobacco [52], and based on well-established economic concepts of price perceptions [53].

To assess perceived cost of SSBs in countries with and without SSB taxes, participants in all four countries in 2017, 2018 and 2019 were asked, “Do drinks with sugar (e.g., Coke) cost more than drinks without sugar (e.g., Diet Coke) in [Australia/Mexico/the UK/the US]?”, with response options ‘No’, ‘Yes – a little more’, ‘Yes – a lot more’, ‘Don’t know’, and ‘Refuse to answer’. The “correct” response was ‘No’ for Australian and US respondents in all three years (aside from US respondents living in cities with an SSB tax), ‘Yes’ for Mexican respondents in all three years, and ‘No’ for UK respondents in 2017 and 'Yes' in 2018–2019.

To assess awareness of SSB taxes in countries that have national or local taxes, participants in Mexico (2017, 2018, 2019); UK (2018, 2019); and US (2019) were asked, “Is there a special tax on sugary drinks in [Mexico/the UK/the US] that makes them more expensive to buy?”, with response options ‘No’, ‘Yes’, ‘Don’t know’ and ‘Refuse to answer’. The “correct” response was ‘Yes’ for Mexican and UK respondents in all available years, and ‘No’ for US respondents overall (aside from US respondents living in cities with an SSB tax).

Participants who responded ‘Yes’ to the tax awareness question above were then asked, “Has the tax changed whether you buy the following drinks for you or your family?” for 14 sugary and non-sugary beverage categories. ‘Regular bottled water’ was only included in waves 2018 and 2019. The beverage categories were described using country-specific wording (e.g., “regular soda or pop” in the US versus “fizzy drinks” in the UK). Response options for each beverage category were ‘Buy less’, ‘Buy more’, ‘No change’, ‘Don’t know’, and ‘Refuse to answer’. To assess a summary of participants’ reported changes in purchases of taxed beverages, we constructed a categorical variable summarizing participants’ responses as ‘Bought less’ (reporting ‘buy less’ for at least one taxed beverage and no ‘buy more’ for any taxed beverage), ‘Bought more’ (reporting ‘buy more’ for at least one taxed beverage and no ‘buy less’ for any taxed beverage), or ‘Mixed responses / No change’ (any other combination of responses across the taxed beverages, including ‘Don’t know’). A parallel variable was constructed for untaxed beverages. Taxed beverage categories were established based on beverage types that would typically be included under SSB taxation schemes, and included regular (e.g., not diet or light) soda, sweetened fruit drinks, regular flavoured waters/vitamin waters, regular sports drinks, and regular energy drinks, which broadly encompass the taxed beverages in all jurisdictions except the City of Philadelphia in the US, where artificially sweetened (“diet”) beverages are also taxed. Untaxed beverages included all remaining categories.

Socio-demographic and SSB perception measures.

Socio-demographic information was collected using measures drawn from government-led national surveys in each country [54–59] and responses were recoded to allow comparison across countries. Socio-demographic variables included age, sex [54], ethnicity [55–58] (recoded to ‘majority group’ or ‘minority group’), education [55, 56, 58, 59] (recoded to ‘low’, ‘medium’ or ‘high’), body mass index (BMI), and subjective income adequacy [60]. An indicator of perceived healthfulness of SSBs (see Table 1) was also examined. Further details on the survey measures and their development are available publicly [61].

**Table 1:** Characteristics of respondents in the International Food Policy Study (weighted), 2017, 2018 and 2019 (*N* = 48,924)

	**Total** ***N* = 48,924**	**Australia** ***N* = 11,588**	**Mexico** ***N* = 11,610**	**UK** ***N* = 12,945**	**US** ***N*= 12,781**
	**% (n)**	**% (n)**	**% (n)**	**% (n)**	**% (n)**
**Year**					
2017	32.3 (15,802)	31.0 (3,595)	32.3 (3,751)	29.4 (3,805)	36.4 (4,651)
2018	35.5 (17,347)	34.0 (3,939)	33.3 (3,869)	40.5 (5,239)	33.6 (4,301)
2019	32.2 (15,775)	35.0 (4,054)	34.4 (3,990)	30.1 (3,901)	30.0 (3,829)
**Age**					
18–29 years	23.7 (11,615)	22.6 (2,618)	30.4 (3,533)	20.2 (2,612)	22.3 (2,852)
30–44 years	28.6 (13,994)	28.6 (3,319)	33.1 (3,838)	26.8 (3,467)	26.4 (3,369)
45–64 years	36.8 (17,983)	36.8 (4,259)	33.7 (3,912)	36.7 (4,751)	39.6 (5,061)
≥ 65 years	10.9 (5,332)	12.0 (1,392)	2.8 (326)	16.3 (2,115)	11.7 (1,500)
**Sex**					
Female	51.3 (25,105)	50.8 (5,890)	52.2 (6,061)	51.3 (6,644)	50.9 (6,510)
Male	48.7 (23,819)	49.2 (5,698)	47.8 (5,549)	48.7 (6,301)	49.1 (6,271)
**Ethnicity **^**a**^					
Majority group	80.4 (39,345)	77.7 (9,003)	82.1 (9,530)	89.5 (11,591)	72.2 (9,222)
Minority group	19.6 (9,579)	22.3 (2,586)	17.9 (2,080)	10.5 (1,355)	27.8 (3,559)
**Education level **^**b**^					
Low	36.1 (17,669)	37.0 (4,292)	19.3 (2,235)	42.3 (5,479)	44.3 (5,663)
Medium	20.8 (10,192)	33.4 (3,874)	12.9 (1,494)	24.0 (3,112)	13.4 (1,713)
High	43.1 (21,063)	29.5 (3,423)	67.9 (7,881)	33.6 (4,354)	42.3 (5,405)
**BMI**					
< 18.5	3.0 (1,462)	3.2 (370)	2.3 (272)	3.1 (400)	3.3 (420)
18.5–24.9	36.0 (17,612)	36.4 (4,213)	40.9 (4,743)	34.0 (4,397)	33.3 (4,258)
25.0–29.9	29.1 (14,219)	27.0 (3,134)	33.4 (3,879)	26.1 (3,380)	29.9 (3,825)
≥ 30.0	20.0 (9,776)	20.8 (2,414)	16.5 (1,914)	16.4 (2,119)	26.0 (3,329)
Missing	12.0 (5,855)	12.6 (1,457)	6.9 (802)	20.5 (2,648)	7.4 (949)
**Income adequacy **^**c**^					
High	69.9 (34,207)	72.4 (8,390)	57.5 (6,675)	75.1 (9,728)	73.7 (9,413)
Low	30.1 (14,717)	27.6 (3,198)	42.5 (4,934)	24.9 (3,218)	26.3 (3,367)
**SSB healthfulness perceptions **^**d**^					
Healthy	10.3 (5,020)	6.0 (690)	16.6 (1,932)	7.3 (942)	11.4 (1,457)
Unhealthy	89.7 (43,904)	94.0 (10,899)	83.4 (9,678)	92.7 (12,003)	88.6 (11,324)
**US city tax status **^**e**^					
Non-tax city					98.9 (8,041)
SSB tax city					1.1 (89)

*UK* United Kingdom, *US* United States, *BMI* body mass index, *SSB* sugar sweetened beverage

^a^ Ethnicity categories as per census questions asked in each country: 1) Australia majority = only speaks English at home, minority = speaks a language besides English at home; 2) Canada majority = White, minority = other ethnicity; 3) Mexico majority = Non-indigenous, minority = indigenous; 4) United Kingdom majority = White, minority = other ethnicity; 5) US majority = White, minority = other ethnicity

^b^ Participants were asked, “What is the highest level of formal education that you have completed?” Responses were categorized as ‘low’ (completed secondary school or less), ‘medium’ (some post-secondary qualifications), or ‘high’ (university degree or higher) according to country-specific criteria

^c^ Participants were asked, “Thinking about your total monthly income, how difficult or easy is it for you to make ends meet?”, with response options ‘Very easy’, ‘Easy’ and ‘Neither easy nor difficult’ categorized as “High”, and ‘Difficult’ and ‘Very difficult’ categorized as “Low”

^d^ Participants were shown a 500 mL bottle of regular soda and asked, “In your opinion, how unhealthy or healthy is this type of drink?”, with response options ‘Very healthy’, ‘Healthy’, ‘A little healthy’ and ‘Neither healthy nor unhealthy’ categorized as “Healthy”, and ‘A little unhealthy’, ‘Unhealthy’ and ‘Very unhealthy’ categorized as “Unhealthy”

^e^ US city/zip code data were only collected in 2018 and 2019

US zip code data were collected in 2018 and 2019. The zip code data were first compared to a database of valid US zip codes to identify and remove any invalid entries. Valid zip codes were then used to construct a variable indicating whether or not respondents lived in any of the seven cities that enforced a municipal SSB tax as of 2018 (‘non-tax city’ vs. ‘SSB tax city’).

### Statistical analyses

Respondents were excluded from the analyses if they had missing data (including ‘Refuse to answer’) for any of the outcome or socio-demographic variables (n = 3,103), excluding BMI, for which missing responses were retained as a valid response category. The final analytical sample consisted of 48,924 respondents across the three years.

Binary logistic regression models were used to assess the odds of participants perceiving drinks with sugar to cost more than drinks without sugar (‘Yes – a little / Yes – a lot’ versus ‘No / Don’t know’) in all four countries, stratified by country.

Binary logistic regression models were also used to evaluate respondents’ awareness of SSB taxes in countries with national or subnational taxes (Mexico, UK, US). These models assessed the odds of participants responding ‘Yes’ versus ‘No / Don’t know’ to whether there is a special tax on sugary drinks in their respective country.

Multinomial logistic regression models were used to evaluate whether participants ‘Bought less’ or ‘Bought more’ (versus ‘Mixed response / No change’) for taxed and untaxed beverage categories, separately for Mexico, the UK, and the US.

Descriptive statistics examined unadjusted percentages for all outcomes of interest, and for responses from participants in US SSB tax cities versus non-tax cities. Results were not disaggregated for participants from Philadelphia (where artificially-sweetened beverages were included in a tax) due to the small number of respondents reporting a Philadelphia zip code.

All descriptive statistics and regression models were run with the post-stratification sample weights applied. Each regression model included variables for year, age, sex, ethnicity, education, income adequacy, and SSB healthfulness perceptions, due to their known associations with dietary patterns and SSB intake [62, 63]. BMI was not included in models due to its high number of missing responses. Models assessing reported changes in beverage purchasing also included a variable for perceived cost of SSBs. 99% confidence intervals were used to account for the large sample size and high number of statistical tests.

## Results

Weighted socio-demographic characteristics of the sample, by country, are presented in Table1. The majority of the sample in each country was female; identified as a majority ethnicity; reported height and weights corresponding to BMIs of 18.5 to 29.9; reported higher income adequacy; and perceived SSBs to be unhealthy. Distribution of education levels varied across countries, with a smaller proportion of participants with “low” education levels in Mexico. In the US, 1.1% (n = 89) of participants reported zip codes corresponding to a city with an SSB tax.

### Perceived cost of SSBs

**Fig. 1:**
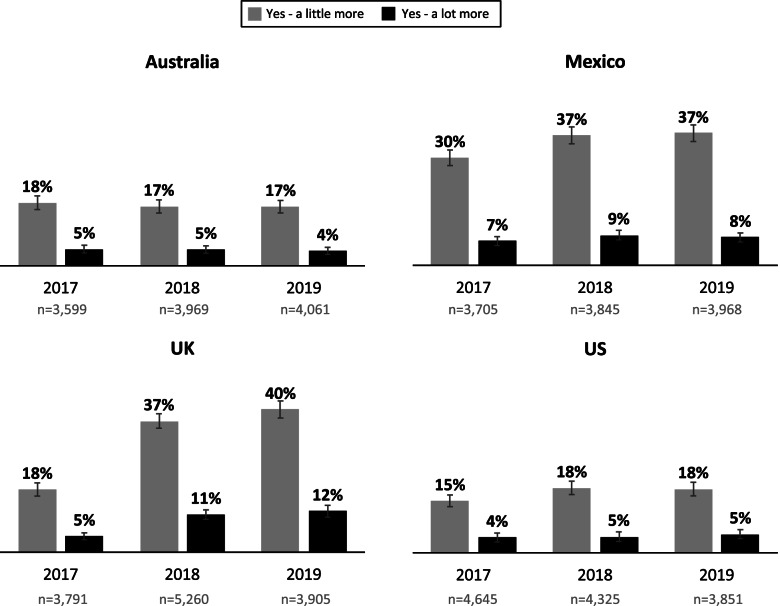
Unadjusted percentages of participants reporting drinks with sugar cost more than drinks without sugar (weighted). *Legend:* Unadjusted percentages of participants in Australia, Mexico, the United Kingdom and the United States reporting that drinks with sugar cost ‘a little’ or ‘a lot’ more than drinks without sugar in 2017, 2018 and 2019 (weighted). Error bars represent 99% confidence intervals

Figure 1 presents the unadjusted percentages of participants in Australia, Mexico, the UK and the US who reported that drinks with sugar cost ‘a little’ or ‘a lot’ more than drinks without sugar in their respective country across 2017, 2018 and 2019. Among respondents who perceived drinks with sugar to cost more than non-sugary drinks, a greater proportion reported they cost ‘a little more’ rather than ‘a lot more’ in all countries. The highest prevalences of perceiving sugary drinks to cost more than non-sugary drinks were observed in Mexico in all three years, and the UK in 2018 and 2019.

Results from the binary logistic regression models investigating perceived cost of SSBs are presented in Table2. In all countries but Australia, there was a higher likelihood of perceiving drinks with sugar to cost more than drinks without sugar in 2018 and 2019 compared to 2017, as well as in 2019 compared to 2018 in the UK. There were no differences in perceived cost of drinks with sugar over time in Australia. Across all countries with and without SSB taxes, the likelihood of perceiving SSBs to cost more than drinks without sugar was higher among the youngest age group and respondents who perceived SSBs as healthy compared to older age groups and those who perceived SSBs as unhealthy. In all countries aside from the UK, perceiving drinks with sugar to cost more than drinks without sugar was higher among male respondents and respondents belonging to a minority ethnicity compared to female and majority ethnicity respondents. UK respondents reporting low education were more likely than those with high education to perceive drinks with sugar to cost more than drinks without sugar, but no other differences across education levels were observed.

**Table 2:** Binary logistic regression models investigating perceived relative cost of beverages with sugar in four countries

	**Australia (*N* = 11,588)**		**Mexico (*N* = 11,610)**		**UK (*N* = 12,945)**		**US (*N* = 12,781)**	
	**‘A little more / A lot more’ **^**a**^		**‘A little more / A lot more’**		**‘A little more / A lot more’**		**‘A little more / A lot more’**	
	**Adjusted Prevalence**	**OR (99% CI)**	**Adjusted Prevalence**	**OR (99% CI)**	**Adjusted Prevalence**	**OR (99% CI)**	**Adjusted Prevalence**	**OR (99% CI)**
**Year **^**b**^								
2017	32.7%	[ref]		[ref]	22.7%	[ref]	22.4%	[ref]
2018	32.1%	0.97 (0.81, 1.16)		1.41 (1.22, 1.63)*	54.9%	4.15 (3.56, 4.83)*	30.0%	1.48 (1.24, 1.77)*
2019	31.0%	0.92 (0.78, 1.10)		1.40 (1.22, 1.62)*	59.5%	5.00 (4.26, 5.88)*	30.9%	1.55 (1.29, 1.86)*
**Age**								
18–29 years	47.3%	[ref]		[ref]	62.0%	[ref]	40.1%	[ref]
30–44 years	39.8%	0.74 (0.61, 0.89)*		0.79 (0.70, 0.90)*	55.7%	0.77 (0.65, 0.92)*	39.1%	0.96 (0.80, 1.15)
45–64 years	24.0%	0.35 (0.29, 0.43)*		0.47 (0.40, 0.55)*	39.4%	0.40 (0.34, 0.47)*	21.9%	0.42 (0.34, 0.51)*
≥ 65 years	20.4%	0.29 (0.21, 0.38)*		0.41 (0.25, 0.68)*	24.2%	0.20 (0.16, 0.24)*	14.8%	0.26 (0.19, 0.35)*
**Sex**								
Female	28.8%	0.75 (0.65, 0.86)*		0.83 (0.74, 0.93)*	43.9%	0.94 (0.83, 1.06)	25.1%	0.77 (0.67, 0.90)*
Male	35.2%	[ref]		[ref]	45.5%	[ref]	30.3%	[ref]
**Ethnicity **^**c**^								
Majority group	25.5%	0.54 (0.45, 0.64)*		0.57 (0.48, 0.67)*	42.3%	0.83 (0.66, 1.03)	20.6%	0.46 (0.39, 0.54)*
Minority group	39.0%	[ref]		[ref]	47.1%	[ref]	35.9%	[ref]
**Education level **^**d**^								
Low	33.2%	1.16 (0.96, 1.40)		1.04 (0.89, 1.21)	47.3%	1.24 (1.08, 1.42)*	29.0%	1.09 (0.93, 1.28)
Medium	32.7%	1.13 (0.95, 1.35)		1.19 (0.98, 1.43)	44.7%	1.11 (0.97, 1.27)	26.7%	0.97 (0.79, 1.19)
High	29.9%	[ref]		[ref]	42.1%	[ref]	27.2%	[ref]
**Income adequacy **^**e**^								
High	30.3%	0.86 (0.73, 1.01)		1.01 (0.89, 1.14)	43.0%	0.87 (0.75, 1.00)	25.9%	0.84 (0.71, 1.00)
Low	33.6%	[ref]		[ref]	46.4%	[ref]	29.3%	[ref]
**SSB healthfulness perceptions **^**f**^								
Healthy	44.6%	2.94 (2.28, 3.80)*		1.23 (1.05, 1.44)*	49.3%	1.45 (1.12, 1.86)*	36.6%	2.30 (1.87, 2.82)*
Unhealthy	21.5%	[ref]		[ref]	40.2%	[ref]	20.1%	[ref]

Results from binary logistic regression models investigating correlates of participants perceiving beverages with sugar to cost ‘a little more’ or ‘a lot more’ versus ‘no different’ than beverages without sugar in Australia, Mexico, the United Kingdom, and the United States

UK, United Kingdom; US, United States; OR, odds ratio; CI, confidence interval; SSB, sugar sweetened beverage

^*^Significantly different (compared to reference group) at *p* < .01

^a^ Participants responding ‘Yes – a little more / Yes – a lot more’ versus ‘No change / Don’t know’ when asked, “Do drinks with sugar (e.g., Coke) cost more than drinks without sugar (e.g., Diet Coke) in [country]?”

^b^ Results for all year comparisons are provided in Additional file 4

^c^ Ethnicity categories as per census questions asked in each country: 1) Australia majority = only speaks English at home, minority = speaks a language besides English at home; 2) Canada majority = White, minority = other ethnicity; 3) Mexico majority = Non-indigenous, minority = indigenous; 4) United Kingdom majority = White, minority = other ethnicity; 5) US majority = White, minority = other ethnicity

^d^ Participants were asked, “What is the highest level of formal education that you have completed?” Responses were categorized as ‘low’ (completed secondary school or less), ‘medium’ (some post-secondary qualifications), or ‘high’ (university degree or higher) according to country-specific criteria

^e^ Participants were asked, “Thinking about your total monthly income, how difficult or easy is it for you to make ends meet?”, with response options ‘Very easy’, ‘Easy’ and ‘Neither easy nor difficult’ categorized as “High”, and ‘Difficult’ and ‘Very difficult’ categorized as “Low”

^f^ Participants were shown a 500 mL bottle of regular soda and asked, “In your opinion, how unhealthy or healthy is this type of drink?”, with response options ‘Very healthy’, ‘Healthy’, ‘A little healthy’ and ‘Neither healthy nor unhealthy’ categorized as “Healthy”, and ‘A little unhealthy’, ‘Unhealthy’ and ‘Very unhealthy’ categorized as “Unhealthy”

Descriptive results from the US (Additional file 1) show that a greater proportion of respondents living in a city with an SSB tax reported that drinks with sugar cost more than drinks without sugar compared to those reporting a zip code with no SSB tax, in both 2018 and 2019.

#### Tax awareness 

**Fig. 2:**
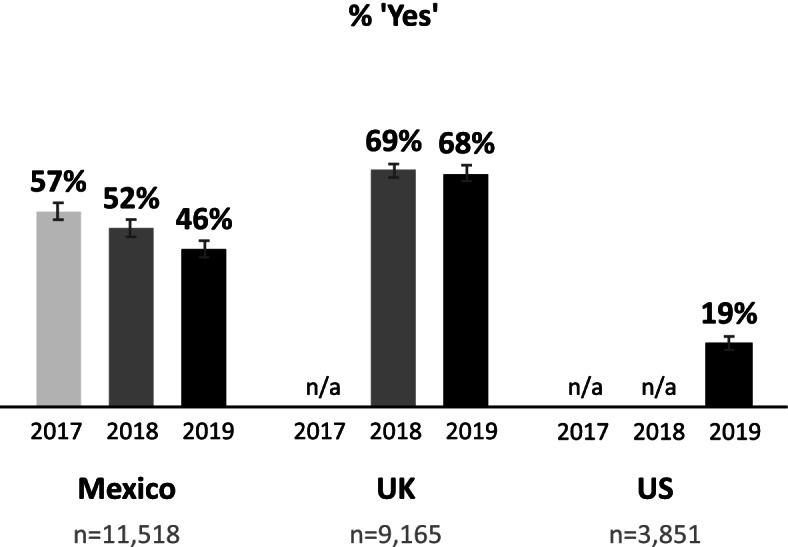
Unadjusted percentages of participants who reported being aware of a tax on sugary drinks (weighted). *Legend:* Unadjusted percentages of participants in Mexico, the United Kingdom and the United States who reported being aware of a special tax on sugary drinks, across available years of data (weighted). Error bars represent 99% confidence intervals. Note: Tax awareness was only queried in countries following the implementation of an SSB tax (2017, 2018 and 2019 in Mexico; 2018 and 2019 in the UK; and 2019 in the US)

The unadjusted percentages of respondents who reported being aware of a special tax on SSBs after taxes were implemented in Mexico (2017, 2018 and 2019), the UK (2018 and 2019) and the US (2019) are presented in Fig. 2. UK respondents’ awareness of their national SSB tax was higher than that in Mexico, and showed no change between 2018 and 2019.

Results from binary logistic regression models assessing awareness of SSB taxes are presented in Table 3. In Mexico, the likelihood of reporting being aware of the national SSB tax was lower with each consecutive year. Younger Mexican respondents were less likely to be aware of the SSB tax compared to those aged 30–64 years, whereas UK respondents aged ≥ 65 years were less likely to be aware of a tax than the youngest respondents. In Mexico and the UK, the likelihood of being aware of a tax was lower among female respondents than males, but there were no differences by sex in the US. UK respondents belonging to a minority ethnicity group were less likely to be aware of an SSB tax compared to majority ethnicities, while the opposite was true in the US. Awareness of a tax in Mexico and the UK was lower among respondents reporting ‘low’ or ‘medium’ education levels than those reporting ‘high’ education levels, and respondents in the UK and US who perceived SSBs as healthy were less and more likely, respectively, to be aware of a tax than those who perceived SSBs as unhealthy.

**Table 3:** Binary logistic regression models investigating awareness of sugar-sweetened beverage taxes in three countries

	Mexico (*n* = 11,610)		UK (*n* = 9,140)		US (*n* = 3,829)	
	**‘Yes’ **^**a**^		**‘Yes’**		**‘Yes’**	
	**Adjusted prevalence**	**OR (99% CI)**	**Adjusted prevalence**	**OR (99% CI)**	**Adjusted prevalence**	**OR (99% CI)**
**Year **^**b**^						
2017	52.0%	[ref]				
2018	47.1%	0.82 (0.71, 0.95)*	60.4%	[ref]		
2019	41.1%	0.64 (0.56, 0.74)*	59.4%	0.96 (0.83, 1.11)	21.7%	
**Age**						
18–29 years	38.5%	[ref]	63.1%	[ref]	25.5%	[ref]
30–44 years	49.0%	1.54 (1.35, 1.75)*	62.6%	0.98 (0.78, 1.22)	24.6%	0.95 (0.64, 1.41)
45–64 years	51.4%	1.69 (1.45, 1.97)*	62.6%	0.98 (0.78, 1.22)	19.1%	0.69 (0.47, 1.02)
≥ 65 years	48.2%	1.49 (0.94, 2.35)	51.1%	0.61 (0.49, 0.77)*	18.0%	0.64 (0.41, 1.00)
**Sex**						
Female	41.3%	0.64 (0.57, 0.72)*	58.2%	0.86 (0.75, 0.99)*	19.6%	0.78 (0.60, 1.02)
Male	52.2%	[ref]	61.7%	[ref]	23.8%	[ref]
**Ethnicity **^**c**^						
Majority group	48.0%	1.11 (0.93, 1.31)	65.5%	1.61 (1.25, 2.08)*	19.1%	0.73 (0.54, 1.00)*
Minority group	45.5%	[ref]	54.1%	[ref]	24.4%	[ref]
**Education level **^**d**^						
Low	41.0%	0.58 (0.50, 0.67)*	58.2%	0.82 (0.70, 0.97)*	22.6%	0.97 (0.74, 1.27)
Medium	44.8%	0.68 (0.56, 0.82)*	58.7%	0.84 (0.72, 0.99)*	19.3%	0.79 (0.57, 1.10)
High	54.5%	[ref]	62.8%	[ref]	23.1%	[ref]
**Income adequacy **^**e**^						
High	47.2%	1.04 (0.92, 1.17)	57.9%	0.85 (0.71, 1.01)	20.8%	0.90 (0.67, 1.22)
Low	46.3%	[ref]	61.9%	[ref]	22.5%	[ref]
**SSB healthfulness perceptions **^**f**^						
Healthy	45.7%	0.92 (0.78, 1.08)	52.5%	0.54 (0.42, 0.71)*	25.2%	1.50 (1.05, 2.14)*
Unhealthy	47.8%	[ref]	67.0%	[ref]	18.4%	[ref]

Results from binary logistic regression models investigating correlates of awareness of sugar sweetened beverage taxes in Mexico, the United Kingdom, and the United States

*UK* United Kingdom, *US* United States, *OR* odds ratio, CI 99% confidence interval, *SSB* sugar sweetened beverage

^*^Significantly different (compared to reference group) at *p* < .01

^a^ Participants responding ‘Yes’ versus ‘No / Don’t know’ when asked, “Is there a special tax on sugary drinks in [country] that makes them more expensive to buy?”

^b^ Results for all year comparisons are provided in Additional file 4

^c^ Ethnicity categories as per census questions asked in each country: 1) Australia majority = only speaks English at home, minority = speaks a language besides English at home; 2) Canada majority = White, minority = other ethnicity; 3) Mexico majority = Non-indigenous, minority = indigenous; 4) United Kingdom majority = White, minority = other ethnicity; 5) US majority = White, minority = other ethnicity

^d^ Participants were asked, “What is the highest level of formal education that you have completed?” Responses were categorized as ‘low’ (completed secondary school or less), ‘medium’ (some post-secondary qualifications), or ‘high’ (university degree or higher) according to country-specific criteria

^e^ Participants were asked, “Thinking about your total monthly income, how difficult or easy is it for you to make ends meet?”, with response options ‘Very easy’, ‘Easy’ and ‘Neither easy nor difficult’ categorized as “High”, and ‘Difficult’ and ‘Very difficult’ categorized as “Low”

^f^ Participants were shown a 500 mL bottle of regular soda and asked, “In your opinion, how unhealthy or healthy is this type of drink?”, with response options ‘Very healthy’, ‘Healthy’, ‘A little healthy’ and ‘Neither healthy nor unhealthy’ categorized as “Healthy”, and ‘A little unhealthy’, ‘Unhealthy’ and ‘Very unhealthy’ categorized as “Unhealthy”

Unadjusted percentages from the US (Additional file 1) suggest that a greater proportion of respondents living in a city with an SSB tax reported that there was a special tax on sugary drinks in the US in 2019, compared to those living in cities without a tax.

#### Reported changes in purchasing behaviour in response to an SSB tax

**Fig. 3:**
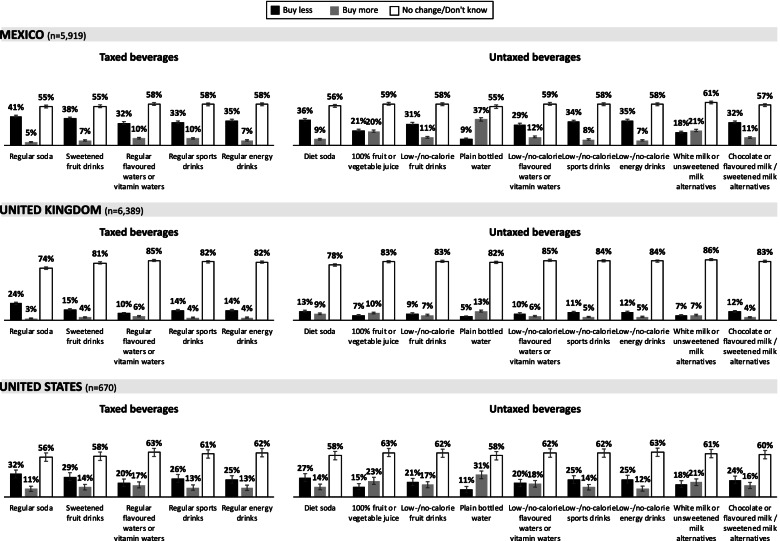
Unadjusted percentages of participants reporting ‘buy less’ or ‘buy more’ taxed and untaxed beverages (weighted). Error bars represent 99% confidence intervals. *Legend:* Unadjusted percentages of participants responding that the SSB tax led them to ‘buy less’ or ‘buy more’ taxed and untaxed beverages, among those who reported being aware of an SSB tax in Mexico, the United Kingdom and the United States, averaged across all available years of data (weighted). Note: Changes in beverage purchasing was only queried in countries following the implementation of an SSB tax (2017, 2018 and 2019 in Mexico; 2018 and 2019 in the UK; and 2019 in the US)

Figure 3 presents the unadjusted percentages of participants who reported that the tax led them to ‘buy less’, ‘buy more’, or ‘no change / don’t know’ for taxed and untaxed beverages, collapsed across all available years of data. Overall, a similar pattern of responses across beverages categories was seen in Mexico, the UK and the US, with the highest magnitudes of change reported by Mexican consumers, followed by the US and the UK. In all three countries, the most common response was ‘no change / don’t know’. However, a substantial proportion of the respondents reported buying ‘less’ of most of the taxed beverage categories and ‘more’ of plain bottled water, while also reporting buying ‘less’ of untaxed beverages such as diet soda, low-/no-calorie sports drinks, and chocolate/flavoured milk. Additional file 2 presents the unadjusted percentages of respondents reporting ‘buy less’ or ‘buy more’ by year, for taxed and untaxed beverages in Mexico, the UK and the US.

Results from multinomial logistic regression models assessing a summary of respondents’ reported changes in taxed beverage purchases, among those who were aware of an SSB tax in their country, are presented in Table 4. Overall, respondents in Mexico were more likely to report buying ‘less’ taxed beverages (versus ‘no change / don’t know’) in 2018 and 2019 compared to 2017. Mexican respondents were also more likely to report buying ‘more’ taxed beverages (versus ‘no change / don’t know) in 2018 and 2019 compared to 2017; however, the proportion of respondents reporting ‘buy more’ was much smaller than those reporting ‘buy less’. The likelihood of buying ‘more’ taxed beverages was lower among participants aged 45–64 years old in Mexico and 45–64 and ≥ 65 years in the UK compared to the youngest respondents. There were no differences by sex in any of the countries, but respondents reporting a majority ethnicity were less likely to report buying ‘more’ taxed beverages than those reporting a minority ethnicity in Mexico and the UK. The probability of buying ‘less’ taxed beverages was higher among respondents who perceived the cost of sugary drinks to be ‘a little more’ than drinks without sugar in Mexico, and those who perceived them to be ‘a little more’ and ‘a lot more’ in the UK, compared to those who perceived no difference in sugary drink prices. The probability of buying ‘more’ taxed beverages was also higher among respondents who reported sugary drinks to cost ‘a little more’ and ‘a lot more’ than drinks without sugar in Mexico, the UK, and the US than those who perceived no price difference. There were further differences by education level, income adequacy, and SSB healthfulness perceptions (Table 4). Parallel results for changes in *un*taxed beverages purchases are presented in Additional file 3.

**Table 4:** Multinomial logistic regression models investigating reported purchase changes in response to a sugar-sweetened beverage tax, among participants who were aware of a tax in their country

	Mexico (*n* = 5,971)				UK (*n* = 6,271)				US (*n *= 712)			
	**Bought less **^**a**^		**Bought more**		**Bought less**		**Bought more**		**Bought less**		**Bought more**	
	**Adjusted Prevalence**	**OR** **(99% CI)**	**Adjusted Prevalence**	**OR** **(99% CI)**	**Adjusted Prevalence**	**OR** **(99% CI)**	**Adjusted Prevalence**	**OR** **(99% CI)**	**Adjusted Prevalence**	**OR** **(99% CI)**	**Adjusted Prevalence**	**OR** **(99% CI)**
**Year **^**b**^												
2017	36.4%	[ref]	5.0%	[ref]								
2018	41.6%	1.32 (1.07, 1.62)*	7.6%	1.74 (1.12, 2.73)*	22.0%	[ref]	4.8%	[ref]				
2019	41.4%	1.29 (1.05, 1.59)*	7.0%	1.58 (1.01, 2.47)*	22.2%	1.02 (0.84, 1.24)	5.5%	1.16 (0.71, 1.89)	21.9%		6.8%	
**Age**												
18–29 years	35.7%	[ref]	8.5%	[ref]	22.9%	[ref]	8.7%	[ref]	15.4%	[ref]	10.7%	[ref]
30–44 years	39.0%	1.13 (0.93, 1.38)	7.1%	0.86 (0.58, 1.28)	18.9%	0.78 (0.58, 1.05)	8.4%	0.92 (0.50, 1.69)	17.3%	1.12 (0.41, 3.02)	8.2%	0.76 (0.26, 2.21)
45–64 years	42.2%	1.21 (0.97, 1.51)	3.4%	0.41 (0.24, 0.69)*	21.9%	0.88 (0.66, 1.17)	3.5%	0.37 (0.18, 0.78)*	25.6%	1.78 (0.67, 4.71)	5.2%	0.53 (0.14, 2.01)
≥ 65 years	42.0%	1.32 (0.69, 2.54)	8.3%	1.09 (0.25, 4.70)	24.3%	1.00 (0.72, 1.38)	2.7%	0.29 (0.11, 0.75)*	30.8%	2.25 (0.74, 6.86)	3.4%	0.36 (0.04, 3.43)
**Sex**												
Female	41.8%	1.14 (0.96, 1.35)	5.4%	0.72 (0.49, 1.08)	22.8%	1.07 (0.88, 1.30)	4.7%	0.84 (0.51, 1.39)	25.3%	1.43 (0.77, 2.67)	4.5%	0.52 (0.18, 1.51)
Male	37.8%	[ref]	7.7%	[ref]	21.4%	[ref]	5.6%	[ref]	18.4%	[ref]	8.9%	[ref]
**Ethnicity **^**c**^												
Majority group	39.9%	0.95 (0.73, 1.23)	4.9%	0.55 (0.35, 0.86)*	19.7%	0.72 (0.50, 1.03)	3.6%	0.44 (0.21, 0.91)*	18.3%	0.62 (0.31, 1.23)	4.6%	0.45 (0.18, 1.12)
Minority group	39.6%	[ref]	8.5%	[ref]	24.4%	[ref]	7.3%	[ref]	25.3%	[ref]	8.7%	[ref]
**Education level **^**d**^												
Low	43.7%	1.39 (1.09, 1.76)*	5.5%	0.81 (0.48, 1.36)	23.9%	1.39 (1.12, 1.73)*	8.1%	2.67 (1.53, 4.64)*	30.0%	1.58 (0.83, 3.01)	5.0%	0.63 (0.25, 1.57)
Medium	40.7%	1.25 (0.93, 1.66)	6.6%	0.94 (0.50, 1.78)	22.8%	1.24 (0.99, 1.55)	4.8%	1.49 (0.85, 2.61)	16.0%	0.70 (0.30, 1.65)	5.9%	0.62 (0.16, 2.43)
High	35.3%	[ref]	7.6%	[ref]	19.5%	[ref]	3.4%	[ref]	20.6%	[ref]	8.6%	[ref]
**Income adequacy **^**e**^												
High	37.2%	0.80 (0.67, 0.95)*	6.6%	0.95 (0.65, 1.41)	19.5%	0.73 (0.58, 0.91)*	5.4%	1.02 (0.59, 1.76)	18.9%	0.74 (0.38, 1.45)	8.2%	1.61 (0.55, 4.72)
Low	42.5%	[ref]	6.3%	[ref]	25.0%	[ref]	4.9%	[ref]	24.7%	[ref]	4.9%	[ref]
**SSB healthfulness perceptions **^**f**^												
Healthy	35.4%	0.73 (0.57, 0.93)*	8.6%	1.64 (1.05, 2.55)*	16.8%	0.55 (0.34, 0.88)*	8.8%	2.76 (1.51, 5.04)*	17.8%	0.65 (0.25, 1.69)	8.9%	1.86 (0.69, 5.01)
Unhealthy	44.3%	[ref]	4.8%	[ref]	28.2%	[ref]	2.9%	[ref]	26.1%	[ref]	4.5%	[ref]
**Perceived cost of SSBs**												
Yes – a little more	41.4%	1.24 (1.03, 1.49)*	7.0%	1.68 (1.11, 2.53)*	20.2%	1.43 (1.14, 1.80)*	6.6%	2.25 (1.18, 4.27)*	18.9%	1.20 (0.56, 2.57)	8.5%	3.64 (1.10, 12.05)*
Yes – a lot more	40.4%	1.21 (0.90, 1.63)	8.2%	1.96 (1.10, 3.49)*	32.4%	2.74 (2.04, 3.67)*	6.0%	2.42 (1.06, 5.56)*	29.2%	2.27 (0.92, 5.60)	11.3%	5.96 (1.61, 22.11)*
No / Don’t know	37.5%	[ref]	4.7%	[ref]	15.6%	[ref]	3.3%	[ref]	17.3%	[ref]	2.6%	[ref]

Results from multinomial logistic regression models investigating correlates of participants in Mexico, the United Kingdom and the United States reporting that they ‘bought less’ or ‘bought more’ taxed beverages (versus ‘mixed response / no change’) in response to a sugar-sweetened beverage tax

UK, United Kingdom; US, United States; OR, odds ratio; CI, confidence interval; SSB, sugar sweetened beverage

^*^Significantly different (compared to reference group) at *p* < .01

^a^ Participants reporting that they ‘Bought less’ (at least one ‘buy less’ and no ‘buy more’ for taxed beverages) or ‘Bought more’ (at least one ‘buy more’ and no ‘buy less’ for taxed beverages) versus ‘Mixed response / No change’ when asked, “Has the tax changed whether you buy the following drinks for you or your family?”

^b^ Results for all year comparisons are provided in Additional file 4

^c^ Ethnicity categories as per census questions asked in each country: 1) Australia majority = only speaks English at home, minority = speaks a language besides English at home; 2) Canada majority = White, minority = other ethnicity; 3) Mexico majority = Non-indigenous, minority = indigenous; 4) United Kingdom majority = White, minority = other ethnicity; 5) US majority = White, minority = other ethnicity

^d^ Participants were asked, “What is the highest level of formal education that you have completed?” Responses were categorized as ‘low’ (completed secondary school or less), ‘medium’ (some post-secondary qualifications), or ‘high’ (university degree or higher) according to country-specific criteria

^e^ Participants were asked, “Thinking about your total monthly income, how difficult or easy is it for you to make ends meet?”, with response options ‘Very easy’, ‘Easy’ and ‘Neither easy nor difficult’ categorized as “High”, and ‘Difficult’ and ‘Very difficult’ categorized as “Low”

^f^ Participants were shown a 500 mL bottle of regular soda and asked, “In your opinion, how unhealthy or healthy is this type of drink?”, with response options ‘Very healthy’, ‘Healthy’, ‘A little healthy’ and ‘Neither healthy nor unhealthy’ categorized as “Healthy”, and ‘A little unhealthy’, ‘Unhealthy’ and ‘Very unhealthy’ categorized as “Unhealthy”

Unadjusted percentages from US respondents (Additional file 1) shows that a greater proportion of participants living in an SSB tax city reported buying less of both the taxed and untaxed beverages, compared to those living in a non-tax city.

## Discussion

In this study we presented new evidence on consumer awareness and responses to SSB taxes from three years of a repeat-cross sectional survey, including differences between countries and across key socio-demographic groups. To summarize, the study found that the perceived costs of drinks with sugar increased from 2017 to 2018 and 2019 in Mexico, the UK and the US, and from 2018 to 2019 in the UK, with no changes in Australia. Awareness of the tax was highest in the UK and did not differ between waves, while there were decreases in awareness of the tax in Mexico in each subsequent wave. A substantial proportion of participants reported an impact of the tax on purchasing taxed (i.e., less healthy) beverages, and this impact was highest in the Mexico.

### Summary of findings & relationship to existing knowledge

Perceived cost of SSBs may play an important role in the relationship between SSB taxes and consumer behaviour. Perceived cost of SSBs relative to drinks without sugar was highest in Mexico across all three years, where a national SSB tax has been implemented since January 2014 and increases in SSB prices relative to non-SSBs were observed [3, 5, 64], and lowest in our ‘control’ country of Australia, where no SSB tax has been implemented. Most importantly, our results demonstrate that perceived cost increased in the UK between 2017 and 2018 following the implementation of their national SSB tax in April 2018 [45], despite the industry-focused nature of the levy and variable pass-through rates observed [24]. Observational evidence thus far on actual price changes in the UK are mixed, with one controlled interrupted time series analysis finding that the prices of some levied beverage categories increased, while others decreased [24]. In a sample of UK parents in 2018, 44% noticed increases in the price of soft drinks as a result of the levy [47].

Overall, in settings with an SSB tax, the proportion of respondents who perceived drinks with sugar to cost ‘a little’ or ‘a lot’ more than non-sugary drinks was reasonably high (approximately half); however, this suggests that raised SSB prices were not salient to about half of all consumers in jurisdictions with an SSB tax. In settings such as the UK, where consumer behaviour change was not a primary objective of the levy, this may be acceptable. In settings where consumer behaviour change is a primary goal (i.e., Mexico and some US cities), the proportion of consumers who did not perceive SSBs to cost more than non-SSBs may be largely made up of less price-sensitive consumers, such as those who do not frequently purchase or consume SSBs, as was suggested in our findings. Notably, almost 20% of respondents in Australia and the US—where no national SSB taxes are implemented—also reported that sugary drinks cost more than drinks without sugar. It is possible that media coverage of international or city-level SSB taxes may have led some participants to attribute higher SSB prices to their country.

The estimates of tax awareness obtained in this study reflect the tax status of the jurisdictions assessed, and are similar to those reported in previous literature. Awareness of a national SSB tax was highest among UK respondents (where the tax had been implemented most recently in 2018), followed closely by Mexico (where a national tax was implemented in 2014), and lowest among respondents in the US, where no national SSB tax is in place. In the US, about three quarters of respondents living in cities with an SSB tax reported being aware of a tax, compared to only 18% among participants in cities without an SSB tax. The proportion of respondents aware of the Mexican SSB tax in this study (57% in 2017, 52% in 2018, 46% in 2019) suggest a gradual decrease in awareness over time, which follows the estimated 65% tax awareness in 2016 reported by Álvarez-Sánchez et al. [36] These decreases in awareness may also suggest that taxes could be increased periodically to maintain their saliency, a practice which has been used successfully in the context of tobacco products [65]. The rates of tax awareness among UK respondents in this study (69% in 2018, 68% in 2019) were lower than those reported by Gillison et al. (92% in 2018) [47]. These differences, however, may be largely attributed to differences in sample profile and data collection methods: the sample recruited by Gillison et al. comprised parents of young children, and respondents were prompted with a full definition of the SDIL prior to being queried about their awareness.

The decrease in tax awareness over time observed in Mexico is in direct contrast to the increases observed for perceived cost of SSBs. The contrast between these two measures may indicate that although awareness of the tax regulation has gradually decreased, the actual price differences between SSBs and non-SSBs remain salient to consumers. Decreased awareness of the Mexican SSB tax may be partially explained by the introduction of more recent nutrition efforts in Mexico (e.g., new front-of-package nutrition labels, television-based healthy eating campaigns [66, 67]), which may have detracted attention from the tax over time, and may also explain some of the differences in magnitude of awareness observed between the UK and Mexico. Given that tax awareness appears to be consistently decreasing in Mexico, and the policy does rely in part on consumer awareness, education campaigns that help to enhance a ‘signalling effect’ and awareness of the tax may be warranted.

The third measure in this study, reported changes in beverage purchasing due to an SSB tax (among participants who were aware of an SSB tax), varied across countries. The pattern of participants buying ‘less’ or ‘more’ across all beverage categories was similar in Mexico, the UK and the US; however, the magnitude of reported changes in response to an SSB tax were largest among the Mexican sample. More modest responses observed in the UK may reflect the industry-focused nature of the SDIL, which encouraged industry reformulation rather than targeting behaviour change by UK consumers [45], as well as the incomplete pass-through of the tax observed in a recent evaluation [24]. In previous studies, approximately 20% of Mexican adults thought the SSB tax was helping to decrease the purchase of SSBs [36]—lower than the 32–41% of respondents in this study stating that the tax led them to buy fewer taxed beverages. In the UK, 41% of a sample of parents expressed intention to reduce SSB consumption for themselves or their family [47], and 71% of a separate sample of UK adults in 2017 believed that the SDIL would be effective [47]. These higher values reported previously (compared to the 10–24% reporting ‘buy less’ for taxed beverages in our study) may again be due to differences in sample profile or question design.

When examining individual beverage categories, a substantial proportion of the respondents reported buying less of most of the taxed beverage categories and more of plain bottled water (this is in contrast to a recent study that found reduced bottled water purchases in the UK following the introduction of the SDIL [68], although this finding may have resulted from a concurrent media focus on the environmental problems of single use plastics), but also reported buying less of untaxed beverages such as diet soda, low-/no-calorie drinks, and chocolate/flavoured milk. Although these results do not necessarily reflect the price mechanisms of the tax, shifting consumers away from artificially-sweetened beverages—whose health effects are still debated [69, 70]—may be a positive outcome. These results also suggest that the majority of consumers are not substituting reductions in sugary drinks with increases in diet drinks, possibly because of concerns about sugar substitutes [71, 72], which reflects patterns of beverage consumption observed in recent years [73].

Across all of the outcome measures, some socio-demographic patterns emerged. Participants who were younger, male, belonging to a minority ethnicity group, and who gave neutral or positive healthfulness ratings to SSBs were more likely to perceive the cost of SSBs to be higher than drinks without sugar, across most or all countries regardless of tax status. Existing evidence suggests that consumers with these characteristics consume SSBs more frequently [74], and thus may be more sensitive to price changes regardless of taxation.

Patterns across socio-demographic groups for tax awareness often contrasted those observed for perceived SSB cost. For example, Mexican respondents who were younger and UK respondents with ‘low’ education were less likely to report awareness of a national SSB tax, despite these groups reporting a higher perceived cost of SSBs relative to drinks without sugar. These groups tend to be more likely to purchase and consume SSBs [74], making them more sensitive to price increases, but may be less aware that the price differences are a result of a tax, possibly due to differing exposure to media sources or government messaging upon tax implementation. Although perceived cost and tax awareness may be linked, they are likely to have unique and separate effects on consumer purchasing behaviour, and may be largely dependent on the extent to which the signalling effects of a tax reach different groups of consumers.

Fewer socio-demographic correlates were identified for reported changes in purchasing behaviour; however, patterns tended to reflect those observed for perceived SSB cost, with a greater proportion reporting that they bought less taxed beverages among participants belonging to a minority ethnicity, reporting ‘low’ education, and indicating lower income adequacy. Perceived cost of SSBs appeared to be a strong influence on reported behaviour change, with higher perception of SSB cost associated with a greater proportion of respondents reporting they ‘bought less’ taxed beverages. These results are in line with previous research suggesting that consumers of lower socioeconomic status are likely to be more responsive to price increases [75].

### Strengths & limitations

Our study represents the first analysis of cost perceptions, tax awareness, and reported purchasing behaviours in response to SSB taxes across multiple countries with and without SSB taxes. However, several limitations should be noted. Respondents were recruited using nonprobability-based sampling; therefore, the findings do not provide nationally representative estimates. For example, although the data were weighted by age, sex, and region, the Mexico sample had higher levels of education than census estimates, while BMI was somewhat lower than national estimates in each of the four countries. The sample of US respondents reporting zip codes from cities with an SSB tax was small, and comparisons to respondents in non-tax cities should be interpreted with caution. Respondent zip code data were not available in 2017; therefore, the authors could not disaggregate results by US city tax status in 2017, which could have influenced perceived cost of SSBs. However, given the small number of US respondents residing in tax cities in 2018 and 2019, this would have been unlikely to have a substantial impact on the overall results. Further, although the acceptable use of self-report methods in diet and nutrition research has been well-established [76, 77], the self-report nature of the survey measures may limit the accuracy of our predictions of changes in purchasing behaviour. In particular, in the UK, the industry-focused nature of the SDIL may have led to some ambiguity among respondents when asked whether there a “special tax on sugar drinks in the UK that makes them more expensive to buy”. In the US, respondents were asked whether there is special tax on sugary drinks ‘in the US’, which some participants—both in cities with and without SSB taxes—may have interpreted as a national-level tax, while others may have interpreted as ‘any’ tax on sugary drinks in the US. Further, substantial reformulation of SSBs in the UK following SDIL implementation [24] (i.e., reductions in sugar content such that previously taxed beverages now fall below the tax threshold) may mean that some ‘taxed’ beverage categories in this study may have included a subset of untaxed beverages in the UK.

### Conclusions & areas for future research

Perceived cost of SSBs and tax awareness were higher in countries where a national SSB tax was present (Mexico, UK), and reported changes in purchasing behaviour were highest in response to Mexico’s national SSB tax. The results suggest that perceived cost and tax awareness represent two distinct constructs, and that there may be room for awareness (of both SSB tax measures and price differentials) to be improved in tax settings where consumer behaviour change is an objective. Increased awareness may be achieved through education campaigns that enhance signalling effects, but must be tailored to each country based on country-specific relationships with taxed beverages. Consumers with characteristics traditionally corresponding to lower socioeconomic status may be less likely to be aware of an SSB tax, but more likely to perceive SSBs to cost more than non-SSBs and respond by reducing their purchasing of those products. Future research should continue to examine tax awareness and responses in countries with SSB taxes to explore why such measures may change over time and across socio-demographic groups.

## Supplementary Information

**Additional file 1**. Unadjusted percentages for all outcomes, among US respondents living in a city with an SSB tax versus without an SSB tax (weighted).

**Additional file 2**. Unadjusted percentages of participants responding that the SSB tax changed whether they buy drinks for themselves or their family, in Mexico (2017, 2018 and 2019) the United Kingdom (2018 and 2019) and the United States (2019). (‘Buy less’ or ‘buy more’ for taxed and untaxed beverage categories, respectively.)

**Additional file 3**. Results from multinomial logistic regression models investigating correlates of participants in Mexico, the United Kingdom and the United States reporting that they ‘bought less’ and ‘bought more’ untaxed beverages (versus ‘mixed response / no change’) in response to a sugar-sweetened beverage tax.

**Additional file 4**. Full year comparisons from binary and multinomial logistic regression models investigating perceived cost of beverages with sugar, awareness of sugar-sweetened beverage taxes, and reported purchase changes in response to a sugar-sweetened beverage tax.

## Data Availability

The datasets used and/or analysed during the current study are available from the corresponding author on reasonable request.
