# Supplementary material for: Tax awareness and perceived cost of sugar-sweetened beverages in four countries between 2017 and 2019: findings from the international food policy study
Source: Int J Behav Nutr Phys Act. 2022 Mar 31;19:38. doi: 10.1186/s12966-022-01277-1 (PMC8973878; doi:10.1186/s12966-022-01277-1)
Supplement: Supplementary file 1 — Additional file 1. Unadjusted percentages for all outcomes, among US respondents living in a city with an SSB tax versus without an SSB tax (weighted). [file 12966_2022_1277_MOESM1_ESM.docx]

**Additional file 1.** Unadjusted percentages for all outcomes, among US respondents living in a city with an SSB tax versus without an SSB tax (weighted)

|  | **SSB tax city ^a^** | **Non-tax city** |
| --- | --- | --- |
|  | **% (n)** | **% (n)** |
| **Do drinks with sugar (e.g., Coke) cost more than drinks without sugar (e.g., Diet Coke) in the US?** |  |  |
| **2018** |  |  |
| Yes – a little more | 40.3% (18) | 18.2% (772) |
| Yes – a lot more | 11.7% (5) | 4.5% (190) |
| No / Don’t know | 48.0% (22) | 77.4% (3292) |
| **2019** |  |  |
| Yes – a little more | 22.8% (10) | 18.0% (682) |
| Yes – a lot more | 14.9% (6) | 5.1% (195) |
| No / Don’t know | 62.4% (27) | 76.9% (2910) |
| **Is there a special tax on sugary drinks in the US that makes them more expensive to buy? ^b^** |  |  |
| Yes | 74.9% (33) | 18.0% (680) |
| No / Don’t know | 25.1% (11) | 82.0% (3106) |
| **Has the tax changed whether you buy the following drinks for you or your family? ^b,c^** |  |  |
| **Taxed beverages ^d^** | 35.5% (12) | 22.6% (153) |
| Bought less | 60.2% (20) | 69.8% (474) |
| Mixed response / No change | 4.3% (1) | 7.7% (52) |
| Bought more |  |  |
| **Untaxed beverages ^e^** |  |  |
| Bought less | 17.5% (6) | 10.8% (74) |
| Mixed response / No change | 73.0% (24) | 77.2% (524) |
| Bought more | 9.5% (3) | 12.0% (81) |
| ^a^ US tax city status only available in 2018 and 2019.  ^b^ Data only available for 2019.  ^c^ Only asked among participants who responded ‘Yes’ to “Is there a special tax on sugary drinks in the US that makes them more expensive to buy?”  ^d^ Participants reporting that they ‘Bought less’ (at least one ‘buy less’ and no ‘buy more’ for taxed beverages), ‘Bought more’ (at least one ‘buy more’ and no ‘buy less’ for taxed beverages), or ‘Mixed response / No change’ (any other combination of responses across the taxed beverage categories).  ^e^ Participants reporting that they ‘Bought less’ (at least one ‘buy less’ and no ‘buy more’ for untaxed beverages), ‘Bought more’ (at least one ‘buy more’ and no ‘buy less’ for untaxed beverages), or ‘Mixed response / No change’ (any other combination of responses across the untaxed beverage categories). | | |
