# Supplementary material for: Tax awareness and perceived cost of sugar-sweetened beverages in four countries between 2017 and 2019: findings from the international food policy study
Source: Int J Behav Nutr Phys Act. 2022 Mar 31;19:38. doi: 10.1186/s12966-022-01277-1 (PMC8973878; doi:10.1186/s12966-022-01277-1)
Supplement: Supplementary file 2 — Additional file 2. Unadjusted percentages of participants responding that the SSB tax changed whether they buy drinks for themselves or their family, in Mexico (2017, 2018 and 2019) the United Kingdom (2018 and 2019) and the United States (2019). (‘Buy less’ or ‘buy more’ for taxed and untaxed beverage categories, respectively.) [file 12966_2022_1277_MOESM2_ESM.docx]

**Additional File 2.** Unadjusted percentages of participants responding that the SSB tax changed whether they buy drinks for themselves or their family, in Mexico (2017, 2018 and 2019) the United Kingdom (2018 and 2019) and the United States (2019). (‘Buy less’ or ‘buy more’ for taxed and untaxed beverage categories, respectively.)


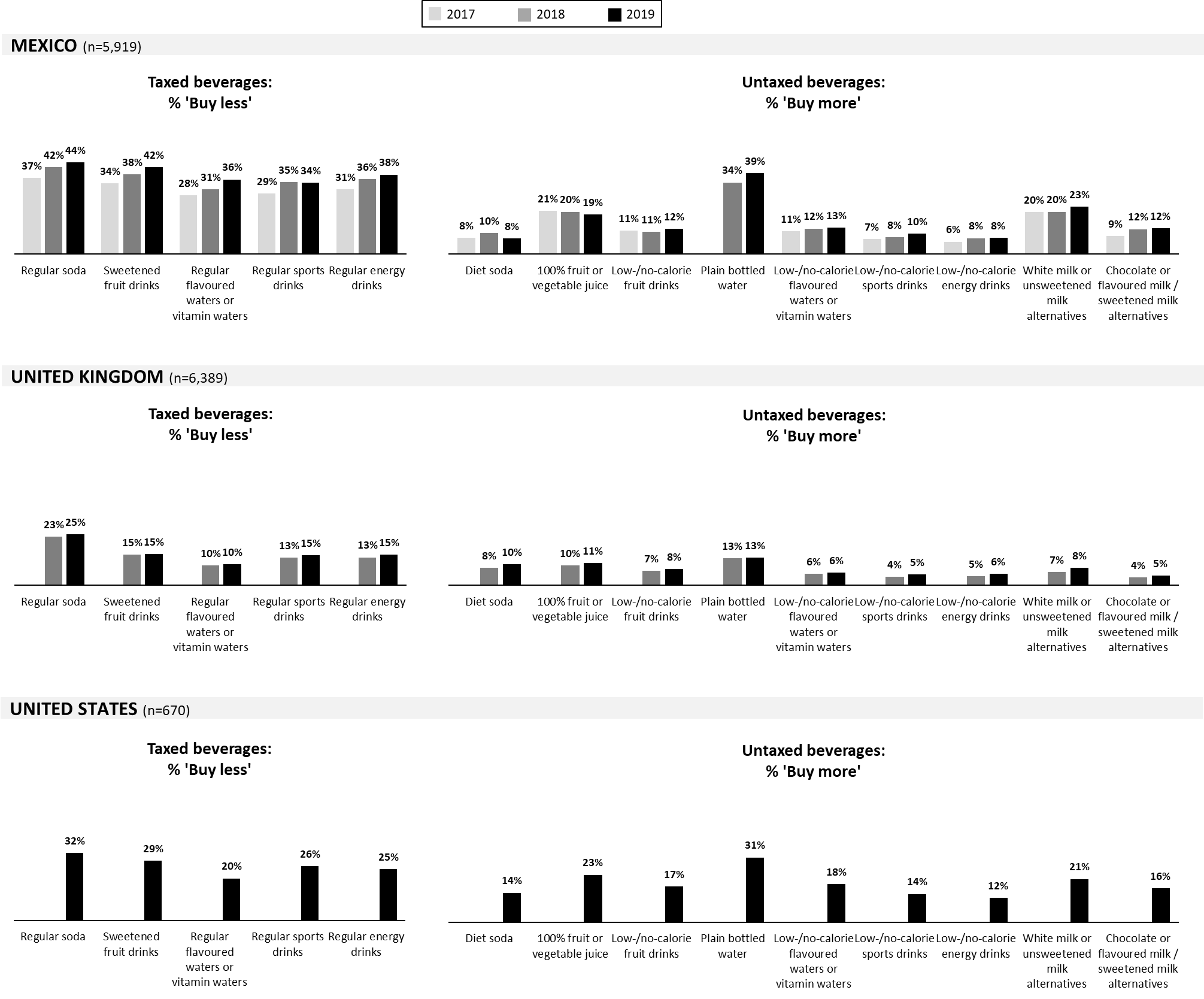


Note: Changes in beverage purchasing was only queried in countries following the implementation of an SSB tax (2017, 2018 and 2019 in Mexico; 2018 and 2019 in the UK; and 2019 in the US).
