# Supplementary material for: Tax awareness and perceived cost of sugar-sweetened beverages in four countries between 2017 and 2019: findings from the international food policy study
Source: Int J Behav Nutr Phys Act. 2022 Mar 31;19:38. doi: 10.1186/s12966-022-01277-1 (PMC8973878; doi:10.1186/s12966-022-01277-1)
Supplement: Supplementary file 3 — Additional file 3. Results from multinomial logistic regression models investigating correlates of participants in Mexico, the United Kingdom and the United States reporting that they ‘bought less’ and ‘bought more’ untaxed beverages (versus ‘mixed response / no change’) in response to a sugar-sweetened beverage tax. [file 12966_2022_1277_MOESM3_ESM.docx]

**Additional File 3.** Results from multinomial logistic regression models investigating correlates of participants in Mexico, the United Kingdom and the United States reporting that they ‘bought less’ and ‘bought more’ untaxed beverages (versus ‘mixed response / no change’) in response to a sugar-sweetened beverage tax.

|  | **Mexico (n=5,962)** | | | | **UK (n=6,271)** | | | | **US (n=712)** | | | |
| --- | --- | --- | --- | --- | --- | --- | --- | --- | --- | --- | --- | --- |
|  | **Bought less ^a^** | | **Bought more** | | **Bought less** | | **Bought less ^a^** | | **Bought more** | | **Bought less** | |
|  | **Adjusted Prevalence** | **OR**  **(99% CI)** | **Adjusted Prevalence** | **OR**  **(99% CI)** | **Adjusted Prevalence** | **OR**  **(99% CI)** | **Adjusted Prevalence** | **OR**  **(99% CI)** | **Adjusted Prevalence** | **OR**  **(99% CI)** | **Adjusted Prevalence** | **OR**  **(99% CI)** |
| **Year ^b^** |  |  |  |  |  |  |  |  |  |  |  |  |
| 2017 | 23.3% | [ref] | 7.4% | [ref] | . | . | . | . | . | . | . | . |
| 2018 | 19.9% | 0.86 (0.67, 1.11) | 11.6% | 1.58 (1.08, 2.30)* | 10.3% | [ref] | 11.6% | [ref] | . | . | . | . |
| 2019 | 19.7% | 0.84 (0.65, 1.08) | 10.5% | 1.40 (0.96, 2.03) | 9.5% | 0.92 (0.68, 1.25) | 12.1% | 1.03 (0.80, 1.33) | 7.3% | . | 15.8% | . |
| **Age** |  |  |  |  |  |  |  |  |  |  |  |  |
| 18-29 years | 17.9% | [ref] | 7.8% | [ref] | 9.0% | [ref] | 14.8% | [ref] | 8.4% | [ref] | 15.7% | [ref] |
| 30-44 years | 20.1% | 1.17 (0.91, 1.49) | 8.5% | 1.13 (0.80, 1.60) | 7.4% | 0.77 (0.48, 1.24) | 11.1% | 0.70 (0.49, 1.01) | 5.5% | 0.60 (0.16, 2.18) | 12.2% | 0.72 (0.26, 1.99) |
| 45-64 years | 20.8% | 1.18 (0.90, 1.55) | 6.3% | 0.82 (0.54, 1.25) | 10.4% | 1.11 (0.70, 1.77) | 10.0% | 0.65 (0.45, 0.93)* | 9.5% | 1.21 (0.36, 4.10) | 19.8% | 1.35 (0.51, 3.56) |
| ≥65 years | 24.2% | 1.78 (0.84, 3.77) | 19.6% | 3.30 (1.34, 8.12)* | 13.6% | 1.54 (0.93, 2.55) | 11.8% | 0.81 (0.55, 1.21) | 6.1% | 0.69 (0.16, 2.93) | 14.8% | 0.90 (0.25, 3.26) |
| **Sex** |  |  |  |  |  |  |  |  |  |  |  |  |
| Female | 21.3% | 1.03 (0.84, 1.28) | 9.4% | 0.94 (0.68, 1.29) | 10.6% | 1.14 (0.84, 1.54) | 11.2% | 0.90 (0.70, 1.16) | 8.7% | 1.48 (0.61, 3.59) | 14.5% | 0.88 (0.41, 1.87) |
| Male | 20.6% | [ref] | 10.0% | [ref] | 9.3% | [ref] | 12.5% | [ref] | 5.9% | [ref] | 16.5% | [ref] |
| **Ethnicity ^c^** |  |  |  |  |  |  |  |  |  |  |  |  |
| Majority group | 19.9% | 0.85 (0.62, 1.17) | 8.8% | 0.78 (0.51, 1.19) | 8.0% | 0.61 (0.36, 1.03) | 11.1% | 0.83 (0.51, 1.35) | 9.4% | 1.61 (0.54, 4.83) | 11.1% | 0.49 (0.24, 1.00) |
| Minority group | 22.1% | [ref] | 10.7% | [ref] | 12.2% | [ref] | 12.5% | [ref] | 5.4% | [ref] | 21.1% | [ref] |
| **Education level ^d^** |  |  |  |  |  |  |  |  |  |  |  |  |
| Low | 23.6% | 1.38 (1.04, 1.84)* | 10.4% | 1.11 (0.71, 1.73) | 11.3% | 1.40 (1.00, 1.95)* | 12.8% | 1.22 (0.92, 1.63) | 11.2% | 1.84 (0.77, 4.39) | 13.3% | 0.76 (0.34, 1.69) |
| Medium | 21.1% | 1.16 (0.82, 1.64) | 8.6% | 0.86 (0.51, 1.47) | 9.9% | 1.19 (0.83, 1.69) | 11.6% | 1.07 (0.81, 1.42) | 5.3% | 0.83 (0.26, 2.66) | 15.5% | 0.84 (0.30, 2.36) |
| High | 18.5% | [ref] | 10.1% | [ref] | 8.6% | [ref] | 11.1% | [ref] | 6.2% | [ref] | 17.7% | [ref] |
| **Income adequacy ^e^** |  |  |  |  |  |  |  |  |  |  |  |  |
| High | 17.9% | 0.69 (0.56, 0.85)* | 10.7% | 1.15 (0.84, 1.59) | 8.2% | 0.66 (0.46, 0.93)* | 12.1% | 1.02 (0.75, 1.38) | 7.1% | 1.05 (0.40, 2.74) | 18.4% | 1.53 (0.62, 3.75) |
| Low | 24.4% | [ref] | 8.7% | [ref] | 11.9% | [ref] | 11.5% | [ref] | 7.3% | [ref] | 12.9% | [ref] |
| **SSB healthfulness perceptions ^f^** |  |  |  |  |  |  |  |  |  |  |  |  |
| Healthy | 19.3% | 0.81 (0.60, 1.11) | 9.7% | 0.96 (0.63, 1.46) | 9.2% | 0.83 (0.42, 1.61) | 11.1% | 0.84 (0.51, 1.40) | 5.8% | 0.75 (0.18, 3.22) | 22.6% | 2.47 (1.05, 5.79)* |
| Unhealthy | 22.7% | [ref] | 9.6% | [ref] | 10.7% | [ref] | 12.6% | [ref] | 8.7% | [ref] | 10.3% | [ref] |
| **Perceived cost of SSBs** |  |  |  |  |  |  |  |  |  |  |  |  |
| Yes – a little more | 21.6% | 1.03 (0.82, 1.29) | 10.2% | 1.17 (0.83, 1.64) | 8.6% | 1.10 (0.78, 1.55) | 13.4% | 2.00 (1.48, 2.70)* | 5.9% | 0.99 (0.32, 3.08) | 17.5% | 1.93 (0.86, 4.36) |
| Yes – a lot more | 20.0% | 0.93 (0.64, 1.34) | 9.9% | 1.11 (0.67, 1.82) | 12.9% | 1.82 (1.17, 2.83)* | 16.4% | 2.69 (1.81, 4.00)* | 9.6% | 1.78 (0.52, 6.11) | 20.8% | 2.52 (0.94, 6.79) |
| No / Don’t know | 21.4% | [ref] | 8.9% | [ref] | 8.4% | [ref] | 7.3% | [ref] | 6.5% | [ref] | 9.9% | [ref] |
| Results from multinomial logistic regression models investigating correlates of participants in Mexico, the United Kingdom and the United States reporting that they ‘bought less’ or ‘bought more’ taxed beverages (versus ‘mixed response / no change’) in response to a sugar-sweetened beverage tax.  UK, United Kingdom; US, United States; OR, odds ratio; CI, confidence interval; SSB, sugar sweetened beverage  *Significantly different (compared to reference group) at p<.01  ^a^ Participants reporting that they ‘Bought less’ (at least one ‘buy less’ and no ‘buy more’ for untaxed beverages) or ‘Bought more’ (at least one ‘buy more’ and no ‘buy less’ for untaxed beverages) versus ‘Mixed response / No change’ when asked, “Has the tax changed whether you buy the following drinks for you or your family?”  ^b^ Results for all year comparisons are provided in Additional File 4.  ^c^ Ethnicity categories as per census questions asked in each country: 1) Australia majority=only speaks English at home, minority=speaks a language besides English at home; 2) Canada majority=White, minority=other ethnicity; 3) Mexico majority=Non-indigenous, minority=indigenous; 4) United Kingdom majority=White, minority=other ethnicity; 5) US majority=White, minority=other ethnicity.  ^d^ Participants were asked, “What is the highest level of formal education that you have completed?” Responses were categorized as ‘low’ (completed secondary school or less), ‘medium’ (some post-secondary qualifications), or ‘high’ (university degree or higher) according to country-specific criteria.  ^e^ Participants were asked, “Thinking about your total monthly income, how difficult or easy is it for you to make ends meet?”, with response options ‘Very easy’, ‘Easy’ and ‘Neither easy nor difficult’ categorized as “High”, and ‘Difficult’ and ‘Very difficult’ categorized as “Low”.  ^f^ Participants were shown a 500mL bottle of regular soda and asked, “In your opinion, how unhealthy or healthy is this type of drink?”, with response options ‘Very healthy’, ‘Healthy’, ‘A little healthy’ and ‘Neither healthy nor unhealthy’ categorized as “Healthy”, and ‘A little unhealthy’, ‘Unhealthy’ and ‘Very unhealthy’ categorized as “Unhealthy”. | | | | | | | | | | | | |
