# Supplementary material for: Tax awareness and perceived cost of sugar-sweetened beverages in four countries between 2017 and 2019: findings from the international food policy study
Source: Int J Behav Nutr Phys Act. 2022 Mar 31;19:38. doi: 10.1186/s12966-022-01277-1 (PMC8973878; doi:10.1186/s12966-022-01277-1)
Supplement: Supplementary file 4 — Additional file 4. Full year comparisons from binary and multinomial logistic regression models investigating perceived cost of beverages with sugar, awareness of sugar-sweetened beverage taxes, and reported purchase changes in response to a sugar-sweetened beverage tax. [file 12966_2022_1277_MOESM4_ESM.docx]

**Additional File 4.** Full year comparisons from binary and multinomial logistic regression models investigating perceived cost of beverages with sugar, awareness of sugar-sweetened beverage taxes, and reported purchase changes in response to a sugar-sweetened beverage tax.

| **PERCEIVED COST OF DRINKS WITH SUGAR** | | | | | | | | |
| --- | --- | --- | --- | --- | --- | --- | --- | --- |
|  | **Australia (N=11,588)** | **Mexico (N=11,610)** | | **UK (N=12,945)** | | **US (N=12,781)** | |  |
|  | **‘A little more / A lot more’ ^a^** | **‘A little more / A lot more’** | | **‘A little more / A lot more’** | | **‘A little more / A lot more’** | |  |
|  | **OR (99% CI)** | **OR (99% CI)** | | **OR (99% CI)** | | **OR (99% CI)** | |  |
| **Year** |  |  | |  | |  | |  |
| 2018 vs. 2017 | 0.97 (0.81, 1.16) | 1.41 (1.22, 1.63)* | | 4.15 (3.56, 4.83)* | | 1.48 (1.24, 1.77)* | |  |
| 2019 vs. 2017 | 0.92 (0.78, 1.10) | 1.40 (1.22, 1.62)* | | 5.00 (4.26, 5.88)* | | 1.55 (1.29, 1.86)* | |  |
| 2019 vs. 2018 | 0.95 (0.80, 1.13) | 1.00 (0.86, 1.15) | | 1.21 (1.05, 1.39)* | | 1.04 (0.87, 1.25) | |  |
| **AWARENESS OF SSB TAXES** | | | | | | | | |
|  |  | **Mexico (n=11,610)** | | **UK (n=9,140)** | | **US (n=3,829)** | |  |
|  |  | **‘Yes’ ^b^** | | **‘Yes’** | | **‘Yes’** | |  |
|  |  | **OR (99% CI)** | | **OR (99% CI)** | | **OR (99% CI)** | |  |
| **Year** |  |  | |  | |  | |  |
| 2018 vs. 2017 | . | 0.82 (0.71, 0.95)* | | . | | . | |  |
| 2019 vs. 2017 | . | 0.64 (0.56, 0.74)* | | . | | . | |  |
| 2019 vs. 2018 | . | 0.78 (0.68, 0.90)* | | 0.96 (0.83, 1.11) | | . | |  |
| **REPORTED PURCHASE CHANGES OF TAXED BEVERAGES** | | | | | | | | |
|  |  | **Mexico (n=5,971)** | | **UK (n=6,271)** | | **US (n=712)** | |  |
|  |  | **Bought less ^c^** | **Bought more** | **Bought less** | **Bought more** | **Bought less** | **Bought more** |  |
|  |  | **OR (99% CI)** | **OR (99% CI)** | **OR**  **(99% CI)** | **OR**  **(99% CI)** | **OR**  **(99% CI)** | **OR**  **(99% CI)** |  |
| **Year** |  |  |  |  |  |  |  |  |
| 2018 vs. 2017 | . | 1.32 (1.07, 1.62)* | 1.74 (1.12, 2.73)* | . | . | . | . |  |
| 2019 vs. 2017 | . | 1.29 (1.05, 1.59)* | 1.58 (1.01, 2.47)* | . | . | . | . |  |
| 2019 vs. 2018 | . | 0.98 (0.80, 1.21) | 0.90 (0.58, 1.40) | 1.02 (0.84, 1.24) | 1.16 (0.71, 1.89) | . | . |  |
| **REPORTED PURCHASE CHANGES OF UNTAXED BEVERAGES** | | | | | | | | |
|  |  | **Mexico (n=5,962)** | | **UK (n=6,389)** | | **US (n=670)** | |  |
|  |  | **Bought less ^d^** | **Bought more** | **Bought less** | **Bought more** | **Bought less** | **Bought more** |  |
|  |  | **OR (99% CI)** | **OR (99% CI)** | **OR**  **(99% CI)** | **OR**  **(99% CI)** | **OR**  **(99% CI)** | **OR**  **(99% CI)** |  |
| **Year** |  |  |  |  |  |  |  |  |
| 2018 vs. 2017 | . | 0.86 (0.67, 1.11) | 1.58 (1.08, 2.30)* | . | . | . | . |  |
| 2019 vs. 2017 | . | 0.84 (0.65, 1.08) | 1.40 (0.96, 2.03) | . | . | . | . |  |
| 2019 vs. 2018 | . | 0.97 (0.75, 1.27) | 0.88 (0.62, 1.26) | 0.92 (0.68, 1.25) | 1.03 (0.80, 1.33) | . | . |  |
| UK, United Kingdom; US, United States; OR, odds ratio; CI, confidence interval; SSB, sugar-sweetened beverage  *p<.01  ^a^ Participants responding ‘Yes – a little more / Yes – a lot more’ versus ‘No change / Don’t know’ when asked, “Do drinks with sugar (e.g., Coke) cost more than drinks without sugar (e.g., Diet Coke) in [country]?”  ^b^ Participants responding ‘Yes’ versus ‘No / Don’t know’ when asked, “Is there a special tax on sugary drinks in [country] that makes them more expensive to buy?”.  ^c^ Participants reporting that they ‘Bought less’ (at least one ‘buy less’ and no ‘buy more’ for taxed beverages) or ‘Bought more’ (at least one ‘buy more’ and no ‘buy less’ for taxed beverages) versus ‘Mixed response / No change’ when asked, “Has the tax changed whether you buy the following drinks for you or your family?”  ^d^ Participants reporting that they ‘Bought less’ (at least one ‘buy less’ and no ‘buy more’ for untaxed beverages) or ‘Bought more’ (at least one ‘buy more’ and no ‘buy less’ for untaxed beverages) versus ‘Mixed response / No change’ when asked, “Has the tax changed whether you buy the following drinks for you or your family?” | | | | | | | | |
